# Prevalence of carotid artery calcification detected by different dental imaging techniques and their relationship with cardiovascular risk factors, age and gender

**DOI:** 10.1186/s12903-023-03564-0

**Published:** 2023-11-30

**Authors:** Tobias Möst, Linus Winter, Yili Elisabeth Ballheimer, Christian Kappler, Magdalena Schmid, Werner Adler, Manuel Weber, Marco Rainer Kesting, Rainer Lutz

**Affiliations:** 1 Department of Oral and Maxillofacial Surgery, University Hospital Erlangen, Glückstraße 11, 91054 Erlangen, Germany; 2 Department of Medical Informatics, Biometry and Epidemiology (IMBE), University of Erlangen- Nuremberg, Erlangen, Germany

**Keywords:** Carotid artery calcification, Atherosclerotic risk factors, Panoramic radiography (PR), Cone beam computed tomography (CBCT)

## Abstract

**Background:**

Atherosclerosis and its secondary diseases display a major threat to patient’s health. Sequelae, like carotid artery calcification (CAC), usually develop over decades and remain asymptomatic for a long time, making preventive measures to reduce mortality and morbidity extremely important. Through panoramic radiography (PR) and cone beam computed tomography (CBCT), dentists may have helpful tools in aiding the holistic care of patients. In this context, the correlation of atherosclerotic risk factors and CAC development have not yet been sufficiently investigated. Thus, the aim of this observational radiological study was to evaluate the diagnostic value of PR compared with CBCT for the detection of CAC in patients older than 60 years. The radiological findings were correlated with gender, age, and cardiac risk factors.

**Methods:**

PRs and CBCTs of N = 607 patients were used for the qualitative analysis and compared. Basic patient information such as age, gender, body mass index (BMI), smoking history as well as patient’s detailed medical history, including heart disease and cardiovascular risk factors such as hypercholesterolemia, arterial hypertension and diabetes mellitus type II were documented and their relation to CAC provided by radiological data was estimated in the form of odds ratios (OR), which were calculated using logistic regression models. Proportions of CAC in different risk groups were compared using Fisher’s exact test, the significance level was set to α ≤ 0.05. The interrater reliability of two physicians was estimated using Cohen’s kappa.

**Results:**

With an accuracy of 90.6%, a sensitivity of 67.5% and a specificity of 99.5% compared to CBCT, PR was a reliable method for the diagnosis of CAC. The overall detection rate for CAC was 27.8% across all age groups. Age (OR: 1.351; p = 0.021), the male sex (OR: 1.645; p = 0.006), arterial hypertension (OR: 2.217; p = < 0.001), heart disease (OR: 1.675; p = 0.006), hypercholesterolemia (OR: 1.904; p = 0.003) and chronic obstructive pulmonary disease (OR: 2.016; p = 0.036) were statistically significant risk factors. When correlated, neither history of stroke nor nicotine abuse showed any statistical significance.

**Conclusions:**

Due to the capabilities of PR in the diagnosis of CAC, dentists can play a vital role in the early diagnosis of vascular disease. Awareness should therefore be raised among dentists regarding the detection of CAC in patients over 60 years of age, with a particular focus on those with arterial hypertension and hypercholesterolaemia.

## Introduction

Atherosclerosis is a chronic degenerative disease of the arterial vasculature characterised by the formation of lipid-containing plaques in the vascular intima [1]. Possible causes of atherosclerosis include chronic endothelial stress, chronic endothelial dysfunction (oxidative stress, nitric oxide deficiency), adaptive intimal thickening and lipid deposition [2, 3]. Atherosclerosis can lead to intimal fibrosis and necrosis of the intima with consequent narrowing and subsequent stenosis of the vessel lumen, to stiffening of the perspective vessel with haemodynamic disturbances, to aneurysm formation due to media atrophy and to acute vessel occlusion due to plaque rupture. In this context, branches of the internal carotid artery, the coronary arteries and the popliteal arteria are predilection sites where atherosclerotic plaques have frequently been observed. Risk factors include hypercholesterolemia, arterial hypertension, nicotine/alcohol abuse, diabetes mellitus and obesity, chronic inflammation [4] and chronic kidney insufficiency [5]. However, atherosclerosis triggers secondary diseases, which are summarised under the term atherosclerotic cardiovascular disease. These include heart attack and stroke, both of which are among the leading causes of death worldwide [6, 7]. The aforementioned sequelae usually develop over decades and remain asymptomatic for a long time, making preventive measures to reduce mortality and morbidity extremely important.

In light of these circumstances, dentists have an important role to play in the prevention of atherosclerotic cardiovascular disease, since the dentist is often consulted, especially by older generations, and dental radiographs, such as the panoramic radiograph (PR), are taken regularly. PRs are standardised, easy to perform, fast, inexpensive and associated with a low radiation dose, making it a convenient tool for follow-up diagnostics.

Arteriosclerotic changes and their potential visibility in the PR were first described as carotid artery calcifications (CAC) by Friedlander and Lande [8]. CACs are usually detected near the angle of the mandible, approximately at the inferior margin of the third cervical vertebra near the hyoid bone, although their location is not limited to the hyoid bone or the thyroid cartilage [9]. However, CAC can be difficult to diagnose because of the potential differential diagnoses/structures that can be seen in the cervical region, such as calcified thyroid cartilage, hyoid bone, calcified styloid ligaments, epiglottis, calcified lymph nodes, phleboliths, sialolithiasis of the submandibular gland, submandibular gland inflammation and tonsil infection [10].

A number of studies have shown that CAC can be detected by PR, with the detected prevalence varying from less than 5% [11, 12] to more than 60% [13]. The large variance in prevalence may be explained by the inclusion criteria chosen. The inclusion of young patients [11, 12] or the study of a small number of patients with significant risk factors [13] result in a prevalence that is not free from bias. Compared to the PR findings, the prevalence of CAC diagnosed by using dental cone beam computed tomography (CBCT) appears to be similar with a rate of 5.7–11.6% and up to 30% or 63% in older populations [14, 15].

According to several study groups, PR is supported as a viable diagnostic aid in the detection of CAC, especially in older patients or high-risk patients for cerebrovascular events [16, 17]. However, it is also widely accepted that for dentists CBCT has a superior diagnostic potential compared to PR due to its three-dimensional evaluation capability allowing for clearer discrimination of anatomical features.

Nevertheless, a clear classification of the diagnostic value of PR in terms of accuracy, sensitivity and specificity compared to CBCT for the diagnosis of CAC is lacking.

In order to assess the diagnostic potential of dentists performing PR and CBCT scans in their daily clinical routine, the aim of this study was to evaluate the diagnostic value of PR compared to CBCT for the detection of CAC in patients older than 60 years presenting to the Department of Oral and Maxillofacial Surgery of University Hospital Erlangen. In addition, the prevalence of CAC was determined and the expected correlation with cardio-/cerebrovascular risk factors was investigated. It was hypothesised that CBCT would have a statistically significantly higher diagnostic value compared to PR and that CAC would be detected significantly more often in patients with a high body mass index (BMI), a positive nicotine history, diabetes mellitus type II, hypercholesterolemia or arterial hypertension.

## Materials and methods

### Study characteristics

The cross-sectional radiological study was conducted at the Department of Oral and Cranio-Maxillofacial Surgery, University Hospital Erlangen, Germany. The study covers an observation period from October 2011 until November 2018. Ethical approval (application no. 555_20 Bc) was obtained from the Ethics Committee of the Medical Faculties of the Friedrich-Alexander University of Erlangen-Nürnberg, and the guidelines of the Declaration of Helsinki were followed. The trial was also registered in the German Clinical Trials Register (DRKS-ID: DRKS00031651) (11/04/2023).

### Inclusion and exclusion criteria

All male and female patients aged 60 years or older who underwent PR and CBCT within one year at the Department of Oral and Maxillofacial Surgery, University Hospital Erlangen, Germany, were included in the study, regardless of the surgical therapy or underlying diagnosis.

Patients were excluded from data analysis if either the PR or the CBCT did not show the region of interest, if the region of interest differed between the PR and CBCT scans, or if one of the modalities performed showed artefacts that made evaluation impossible.

### Outcome variables and hypothesis

The primary outcome variable of the study was to determine and to evaluate the ability (accuracy, sensitivity, specificity) of panoramic radiography compared with CBCT to detect CAC.

The secondary outcome variable was to determine the prevalence of CAC and to verify whether the patient age and gender were related to an increased risk of CAC.

The third outcome variable was to test whether a relationship between the detected CACs and underlying cardiac disease and/or cardiovascular risk factors, such as BMI, smoking habit, hypercholesterolemia, arterial hypertension and diabetes mellitus type II could be established. The null hypothesis is that the risk of CAC, is independent of patient age, sex, underlying cardiac disease and/or cardiovascular risk factors.

### Radiological analysis and measurements

**Fig. 1:**
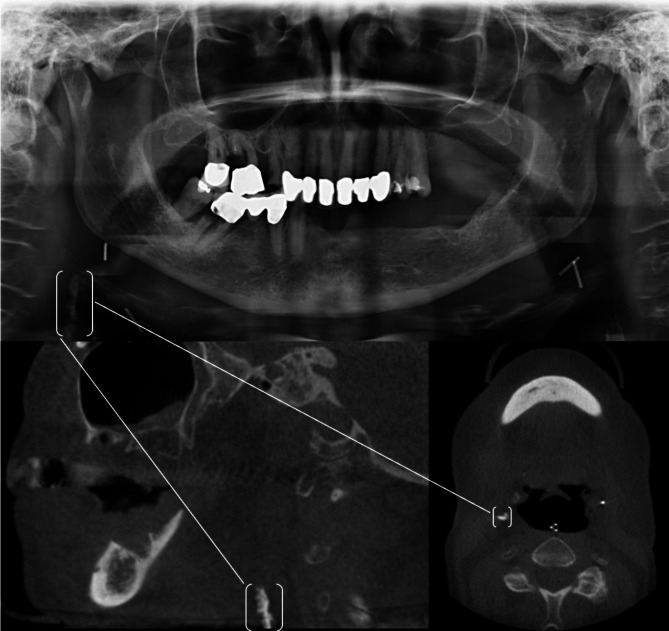
64-year-old female patient with a BMI of 28. At the time of recording, the patient’s medical history listed risk factors included arterial hypertension, other heart diseases, as well as hypercholesterolemia

**Fig. 2:**
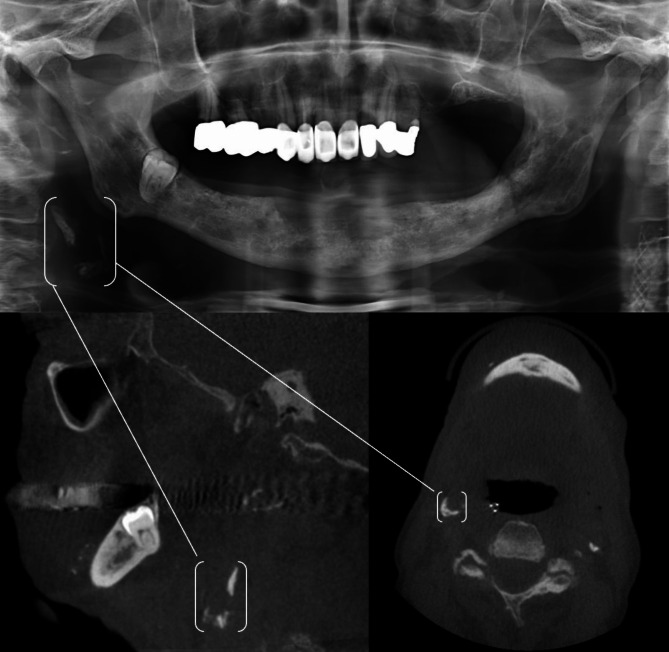
71-year-old male patient with a BMI of 20. Apart from nicotine abuse, no other risk factors were documented

**Fig. 3:**
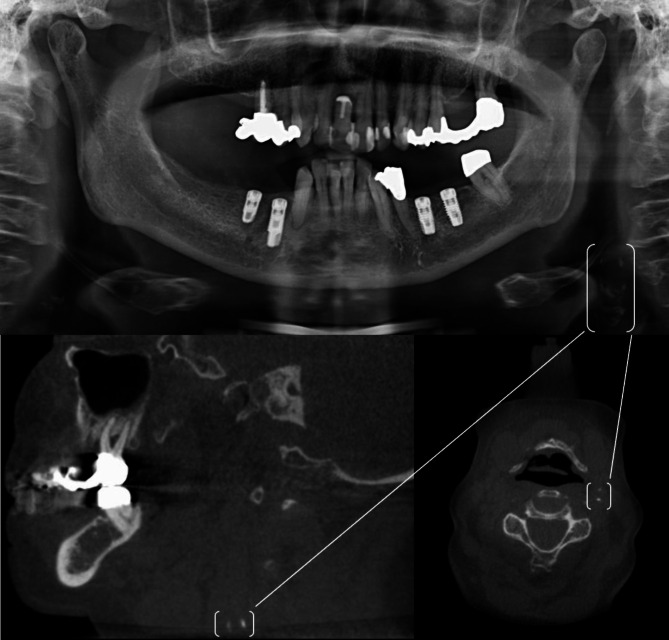
78-year-old female patient. Arterial hypertension and diabetes mellitus type II were documented as risk factors in the patient’s medical history

Standardised digital PR (Sirona Orthophos XG; settings: exposure time = 14.1 s, 64 kV, 16 mA; Sirona Dental Systems GmbH, Bensheim, Germany) and CBCT scans (3D eXam®; settings: exposure time = 4s, 120 kV, 5 mA, resolution = 0.3 voxel; KaVo Dental, Biberach, Germany) were performed at the Department of Oral and Cranio-Maxillofacial Surgery, University Hospital Erlangen, according to the manufacturers’ positioning and exposure protocols. Radiographs were managed in the SIDEXIS XG database and software and saved in TIFF format for further analysis. CBCT files were managed in the eXamVision database and software as DICOM files for further analysis. CACs were characterised as a radiopaque nodular mass adjacent to the cervical vertebrae in the region of the C3–C4 intervertebral disc level or near the retromolar level, independent of the hyoid bone, according to Friedlander and Lande [8].

(Figs. 1, 2 and 3**)**.

The analyses of all CBCTs and PRs were performed by two experienced raters. Both raters were trained for two weeks in the use of the above parameters on 20 PRs and 20 CBCTs: 10 from patients with CAC and 10 from patients without CAC. Discrepant results were discussed to increase reliability. After calibration, all data were analysed by both raters individually in a double-blind design with an interval of 14 days between the first and second assessment.

### Statistical analysis

To compare PR and CBCT, accuracy, sensitivity, and specificity were calculated using CBCT as gold standard. Interobserver agreement between two raters was examined using Cohen’s kappa. Kappas were compared with the Z-test. Odds ratios were calculated using logistic regression models to examine the relation between CAC as dependent variable and age, gender, arterial hypertension, hypercholesterolemia, diabetes mellitus type II, chronic pulmonary disease, stroke, and other heart diseases as independent variables in bivariable models including only one risk factor. In a multivariable model, the relation between CAC and arterial hypertension, other heart disease, stroke, diabetes mellitus type II, chronic obstructive pulmonary disease, nicotine abuse and hypercholesterolemia was examined. Simultaneous presence of arterial hypertension and hypercholesterolaemia was examined in a last model without further risk factors. Fisher´s exact test was used in order to compare CAC prevalence in different age groups and body weight groups. A p-value of less than 0.05 was considered to be significant. The statistical analysis was performed using the publicly available statistical software “R” V4.1.2 [18], to calculate kappa we used the library psych [19].

## Results

### Characterisation of study population

**Fig. 4:**
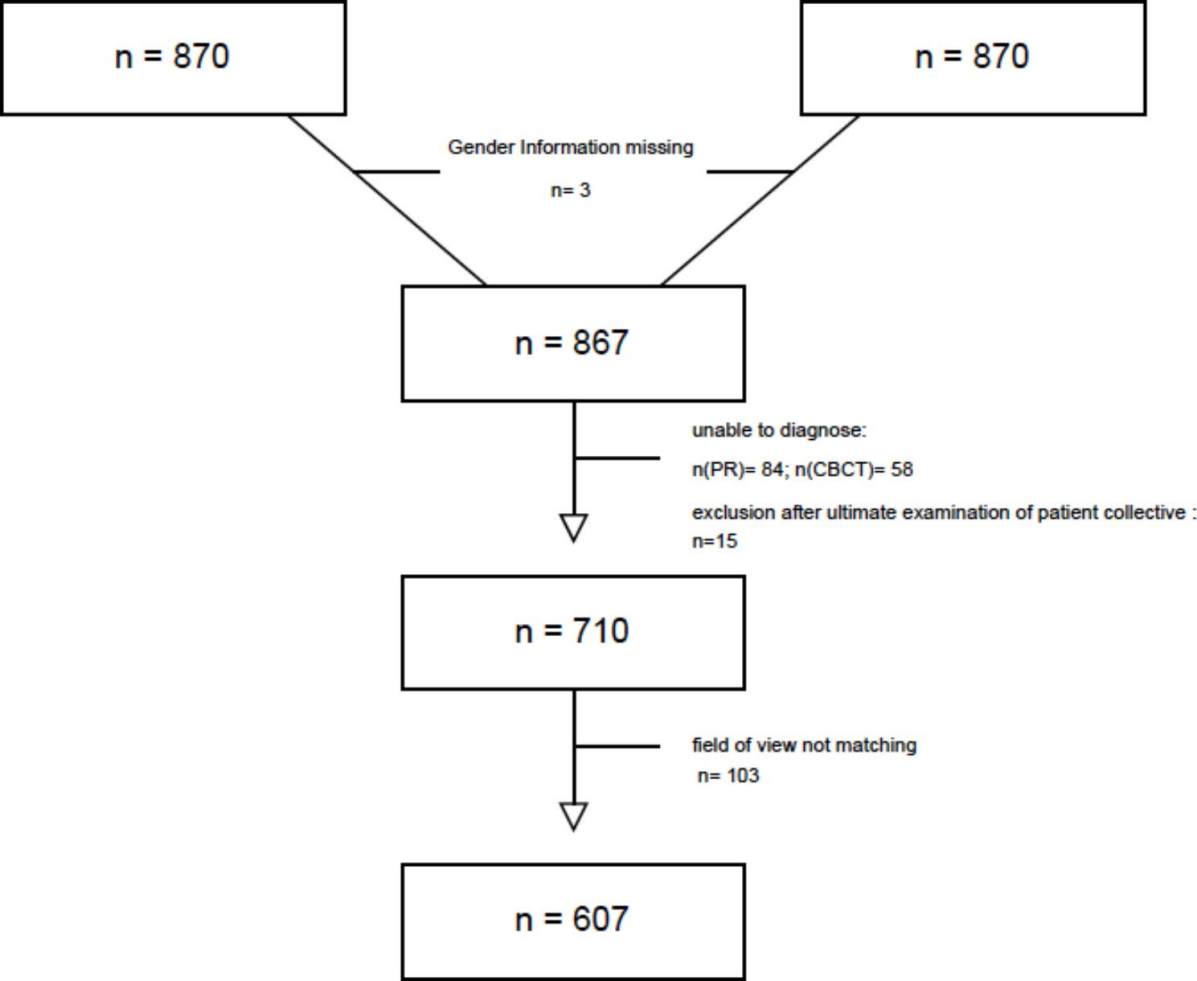
Flow diagram showing the final study population

After initial screening, N = 20,770 PRs and N = 1,863 CBCTs were found that met the initial study criteria. After excluding PRs/CBCTs that were not within 1 year of each other and excluding multiples, N = 870 PRs/CBCTs remained. N = 84 PRs and N = 58 CBCTs were excluded due to severe bias, missing digital data or other factors that made it impossible to review the recorded file. Thus, N = 710 patients met the study criteria. However, on further review, N = 103 patients had to be discarded due to defined exclusion criteria. In N = 55 patients, the CBCT did not show the cervical region represented by the PR. In N = 48 patients, the PR did not show the cervical region represented by the CBCT. In conclusion, a total of N = 607 patient PRs/CBCTs were further analysed and statistically evaluated in this study **(**Fig. 4**).**

Within the study population, N = 320 patients were female (52.7%) and N = 287 were male (47.3%). The mean age of the study population was 70.2 years (± 6.9 years). The mean BMI was 25.7 (± 4.5). In total, arterial hypertension was documented in N = 384 patients (63.3%), diabetes mellitus type II in N = 108 (17.8%), hypercholesterolemia in N = 339 (55.8%), chronic obstructive pulmonary disease in N = 40 (6.6%), smoking habits in N = 157 (25.9%), stroke in N = 71 (11.7%) and other cardiac diseases in N = 244 (40.2%) patients.

### Inter-rater variability

The inter-rater reliability between the two raters was determined using Cohen’s kappa. Furthermore, agreement was achieved when both raters diagnosed the underlying CBCT/PR as CAC (+) or CAC (-) identically. For PR, agreement was 96.5% with a Cohen’s kappa of 0.844 (95% CI: 0.807–0.881). The agreement for CBCT was 95.6% with a Cohen’s kappa of 0.864 (95% CI: 0.836–0.893). Both kappa values did not differ significantly (p = 0.401).

### Diagnostic quality of PR compared to CBCT

When the results of PR were compared with the data generated by CBCT, an accuracy of 90.6% with a sensitivity of 67.5% and a specificity of 99.5% could be established, accepting CBCT as a gold standard diagnostic modality.

### Age-dependent prevalence of CAC

**Fig. 5:**
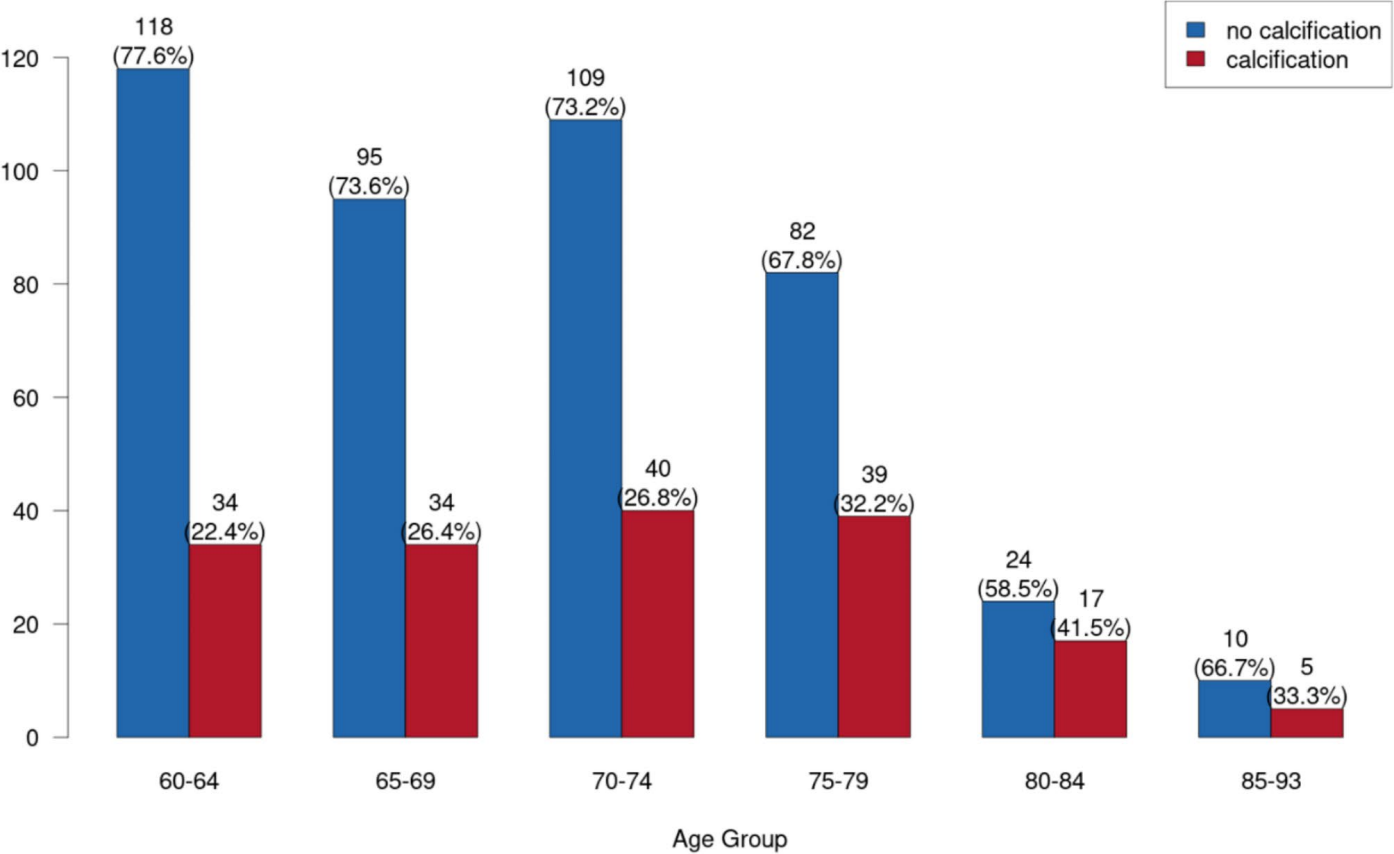
Age-dependent prevalence of CAC. Blue columns = no calcification; red columns = calcification

CACs were detected in the study population aged 60–64 years in N = 34 (22.4%), in the study group aged 65–69 years in N = 34 (26.4%), in the study group aged 70–74 years in N = 40 (26.8%), in the study group aged 75–79 years in N = 39 (32.2%) and in the study group 80–84 years for N = 17 (41.5%) patients. In the group of patients 85 years or older, CAC could be evaluated in N = 5 (33.3%) cases **(**Fig. 5**)**. When comparing the prevalence’s between these groups, no statistically significant difference was found (p = 0.172). However, increasing age (10-year interval) was a statistically significant odds for CAC formation (OR: 1.351, 95% CI 1.047; 1.745; p = 0.021). The odds of developing CAC were increased by 35.1% for ten-year older patients.

For the entire study population, the prevalence of CAC was 27.8% (169 CAC-positive patients).

### Body-weight-dependent prevalence of CAC

**Fig. 6:**
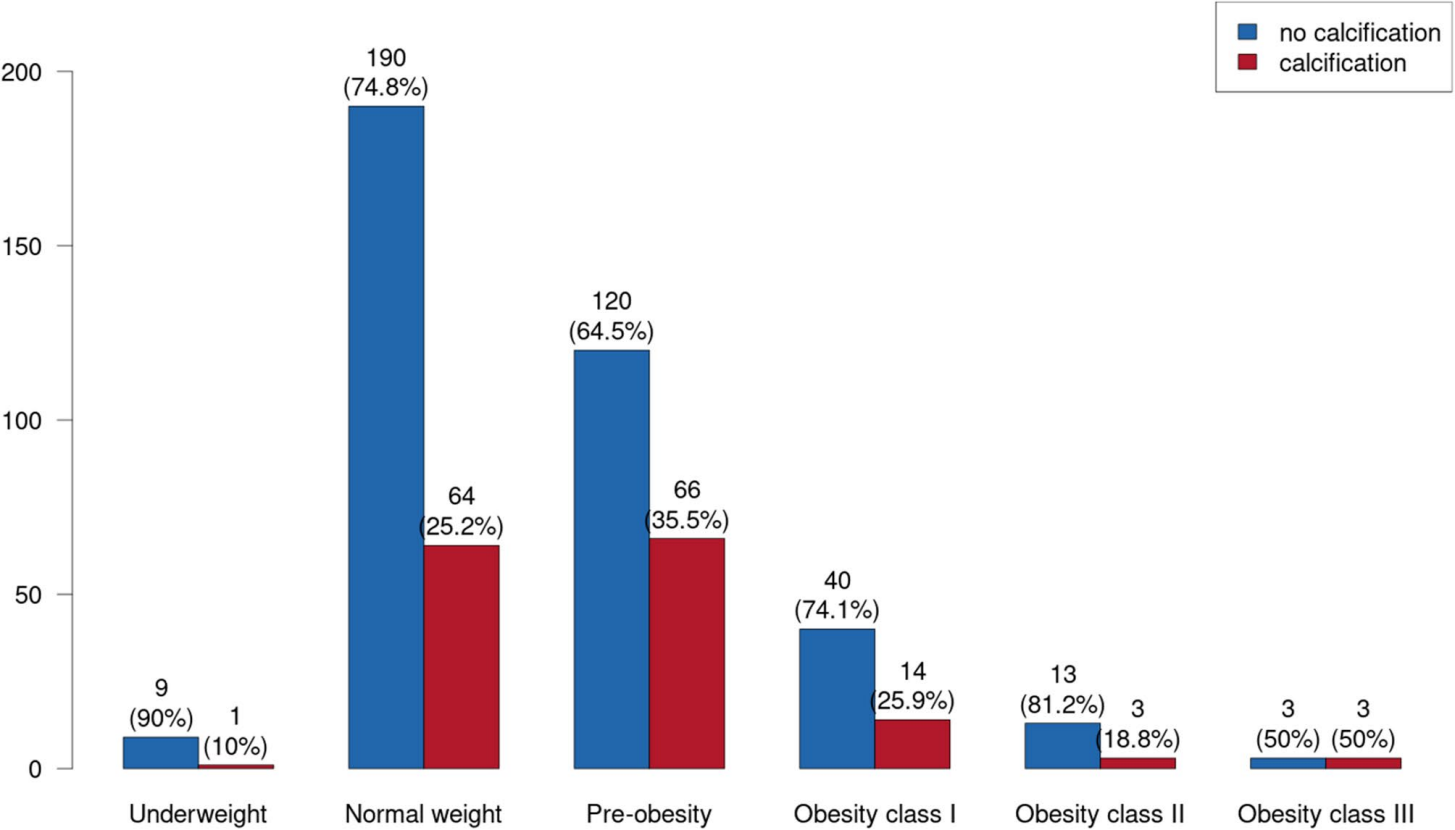
Body-weight-dependent prevalence of CAC. Blue columns = no calcification; red columns = calcification

Underweight patients had a CAC prevalence of 10%. In contrast, normal-weight patients had a CAC prevalence of 25.2%. A higher prevalence of CAC was found in “pre-obese” patients with a rate of 35.5%. For higher body weights, lower prevalence rates were found (obesity class I: 25.9%; obesity class II: 18.8%). However, the highest prevalence rate of 50% was detected for obesity class III. Neither body weight (p = 0.086) nor obesity (p = 0.682) is a statistically significant risk factor for CAC **(**Fig. 6**)**. BMI was not statistically associated with the prevalence of CAC (OR: 1.028; 95% CI: 0.987; 1.071; p = 0.185).

### Correlation of other potential risk factors with CAC formation

In our study population, the risk of CAC formation was statistically correlated with arterial hypertension (OR: 2.217; 95% CI 1.476; 3.392; p < 0.001), other heart diseases (OR: 1.675; 95% CI 1.164; 2.415; p = 0.006), diabetes mellitus type II (OR: 1. 707; 95% CI 1.092; 2.644; p = 0.017), hypercholesterolemia (OR: 1.904; 95% CI 1.266; 3.028; p = 0.003), chronic obstructive pulmonary disease (OR: 2.016; 95% CI 1.034; 3.864; p = 0.036) and male sex (OR: 1.645; 95% CI 1.152; 2.357; p = 0.006).

History of stroke (OR: 1.283; 95% CI 0.742; 2.167; p = 0.359) and nicotine abuse (OR: 1.308; 95% CI 0.873; 1.946; p = 0.188) were not statistically associated with the prevalence of CAC.

On multivariate analysis, arterial hypertension (OR: 1.666; 95% CI 1.017; 2.771; p = 0.045) and hypercholesterolemia (OR: 1.649; 95% CI 1.047; 2.64; p = 0.033) remained statistically significant risk factors, while other cardiac disease, stroke, diabetes mellitus type II, chronic obstructive pulmonary disease, and nicotine abuse were not significant. The relative risk of CAC formation is increased by 66.6% for arterial hypertension and by 64.9% for hypercholesterolaemia. The OR increases to 2.221 (95% CI 1.502; 3.306; p < 0.001) in cases of simultaneous arterial hypertension and hypercholesterolaemia.

## Discussion

The ever-evolving field of dentistry is becoming increasingly specialised in all its fields, such as oral surgery, restorative dentistry, periodontics, prosthodontics, orthodontics, but many more. However, all disciplines require knowledge and consideration of anamnestic conditions, such as the presence of underlying internal diseases, the associated long-term medications and their influence on the stomatognathic system.

Provided that the dentist has the knowledge, skills and ability to recognise the pathophysiological conditions of the stomatognathic system, he or she can play an essential role in the holistic care of the patient, in terms of initiating further diagnosis and treatment of compromising internal diseases. In this context, it has been shown that radiological signs of secondary hyperparathyroidism [20] or reduced bone mineral density [21] can be detected by PR. Focusing on the diagnosis of CAC, with its threatening morbidity and mortality, follow-up data underline the potential risk of CAC diagnosed by PR. Long-term follow-up data have shown that the presence of CAC in the PR is associated with stroke and/or ischaemic events and decreased survival [16]. Other studies with shorter follow-up underline an increased odds ratio for events of myocardial infarction, stroke, transient ischemic attack, revascularisation and angina in patients with CAC detected in the PR [22–24]. In order to assess the reliability of PR compared to CBCT as accurately as possible, the primary aim of the study was to describe the accuracy of PR compared to CBCT by comparing the generated data of 607 patients. Our data showed that PR is a method for the detection of CAC with an accuracy of 91.6%, a sensitivity of 67.46% and a specificity of 99.5%. These findings ascertain the fact, that the dentist can play a relevant role in the holistic care of the patient. Although incidental, early findings of CAC during routine dental appointments might improve the long-term survival of patients through early referral and interventions to/of specialists. Our findings also support the perspective goal of sensitising fellow dentists as well as dental students to the possible presence of CAC in PR, in order to take full advantage of the PR diagnostic values. As atherosclerosis is a common disease of the ageing population, the secondary outcome variable was to evaluate the prevalence of CAC in a group of patients treated by the Department of Oral and Maxillofacial Surgery of University Hospital Erlangen aged 60 years or older. The evaluation showed CACs in 169 patients (27.8%), with an increasing prevalence in the older age groups. In this context, the prevalence appears to decrease in the group of patients over 85 years of age, although this study group is the smallest (N = 15) and therefore the power of the data may be limited. Furthermore, it could be hypothesised that this study group only reached this age due to the lack of arteriosclerotic changes and associated morbidity. Regarding the age-dependent prevalence of CAC in PR, several studies have attempted to describe the prevalence, but most have methodological limitations. It seems that older patients have an increased likelihood of CAC in PR [10, 25, 26], although it should be noted that Santos et al., Ertas et al. and Bilgin Cetin et al. included relatively young patients (Santos et al. >18 years [26], Ertas et al. >40 years [10], Bilgin Cetin et al. <40 years [25]). It is expected that the observed age dependence is due to a wide age range and could be reduced by narrowing it. To describe the prevalence of CAC as accurately as possible, we divided our study population into six age groups.

The subdivision allows an age-dependent description of CAC presence and shows an increase of 35.1% over a 10-year interval. In addition to patient age, the presence of other atherosclerotic risk factors and their association with CAC should be evaluated. In our manuscript, several underlying diseases were collected and correlated with CBCT findings. For example, Janiszewska-Olszowska et al. [27] were able to describe the prevalence of CAC, but a precise risk factor assessment could not be performed due to limited access to medical records. As a result, or for other reasons, underlying medical conditions are often neglected and only the presence of CAC is examined [11, 26–28]. Studies attempting to analyse a possible correlation of risk factors with CAC have methodological weaknesses (young patient population). For example, Cetin et al. could not find a significant correlation with an increased risk of carotid calcification apart from age [25]. Nevertheless, Ertas et al. found that higher rates of CAC were reported in patients with cerebrovascular accidents and elevated cholesterol levels [10]. To increase the soundness of our study, a detailed risk factor analysis was performed in our patient group. In this context, the highly statistically significant correlation of other cardiac diseases, diabetes mellitus type II, arterial hypertension and hypercholesterolaemia was surprising. On the other hand, history of stroke and nicotine abuse did not correlate significantly with CAC prevalence, which was expected. The statistically significant association with gender and the lack of association with body weight were not expected. From a methodological point of view, the study aimed to generate data of high validity. For this reason, in contrast to other studies such as Bilgin Cetin et al. [25] or Janiszewska-Olszowska et al. [27], all radiographs and tomographs were reviewed independently by two blinded raters. The raters had to meet the previously established eligibility criteria. Blinding to previous assessments of the same patients, PR/CBCT, age, sex, medical conditions or the results of the second rater avoided multiple sources of potential bias, thereby ensuring the highest quality of assessment.

Nevertheless, a number of methodological difficulties were encountered in generating the data, and hence reliable results. For each patient dataset analysed in this study, the main objective remained to correctly identify CACs as true positives, while minimising false negatives and false positives. In order to minimise errors in PR assessment, graders had to take into account that the forced perspective of a 2D rendered image from a 3D space resulted in overlapping anatomical structures, e.g. the triticeous cartilage/spine overlapping the CAC, which are the most common reasons for false positives. Reproducible interpretation requires reliable, standardised data generation, with the medical assistants involved in image acquisition also playing an essential role in the diagnosability of CAC in PR/CBCT. The positioning of the patient in the PR/CBCT scanner has a significant impact on the resulting image, especially in PR scanners. For example, images of patients that are not properly aligned can result in images in which anatomical features are distorted, one side of the patient is not adequately imaged, or images with the aforementioned superimposed structures are produced. Another potential limiting factor is the number of patients included. With more than 600 patients, the number in this study is high, but the question remains whether the evaluated prevalence generated by the included number represents the whole population. Furthermore, it should be emphasised that the aim of the study was to compare different dental radiological methods for the diagnosis of CAC in order to emphasise the potential of dentists for holistic patient care. Due to its three-dimensional detection potential, CBCT has the highest diagnostic potential of all dental radiological methods, although in this study CBCT was chosen as the gold standard, knowing that ultrasound is the optimal diagnostic method for CAC diagnosis. Recognizing this pitfall, further studies would be of interest, in which CBCT/PR CAC findings are subsequently correlated with carotid ultrasound CAC detection.

Summarising the results of the study, it can be stated, that aside of CBCT, PR is a possible diagnostic tool for the detection of CAC, especially in patients with multiple risk factors for atherosclerosis. As a result, the dentist can play an important role in the holistic care of the patient and in the prolongation of the patient’s life. If the dentist detects a previously unknown CAC, referral to a general or internal medical physician should be initiated/recommended. The dentist must be aware of the pathogenesis and the potential risk of stroke with high morbidity and mortality. The educational implication of this study is that the diagnosis of CAC should be included in student training.

## Data Availability

The data and materials collected in this research are available from the corresponding author when requested reasonably.
